# Frequency of canine nt230(del4) MDR1 mutation in prone pure breeds, their crosses and mongrels in Israel - insights from a worldwide comparative perspective.

**DOI:** 10.1186/s12917-017-1251-9

**Published:** 2017-11-13

**Authors:** Yaron Dekel, Yossy Machluf, Aviad Stoler, Arava Aderet, Daniel Baumel, Efrat Kellerman, Yoram Plotsky, Oshrat Noked Partouche, Gal Elhalal, Izhar Ben-Shlomo, Dani Bercovich

**Affiliations:** 1 Shamir Research Institute, University of Haifa, P.O.Box 97, 1290000 Kazerin, Israel; 2 Zefat Academic College, Zefat, Israel; 3 Tel Hai College, 12210 Tel-Hai, Israel; 4 GGA – Galil Genetic Analysis Ltd, P.O.Box 3664, 12900 Kazerin, Israel; 5 Specialist in the Fields of Genetics, Epidemiology and Molecular Biology, Rehovot, Israel; 6 Vetmarket Ltd. Industrial Park Hevel Modiin, P.O.Box 960, 6085001 Shoham, Israel; 7 Faculty of Medicine in the Galilee, Bar-Ilan University & Baruch Padeh Medical Center, Poria, Israel

**Keywords:** nt230(del) MDR1 mutation, Macrocyclic lactones, Sheepdogs, Drug sensitivity, Taqman assay

## Abstract

**Background:**

Sensitivity to macrocyclic lactones, which are commonly used in veterinary clinics, was first found in Rough Collies, and was attributed in 2001 to a 4 bp deletion in the MDR1 gene. The list of affected breeds currently includes 13 breeds. Researchers from different countries and continents examined the allelic frequencies of the nt230(del4) MDR1 mutation, emphasizing the clinical importance of this test not only to mutation-prone dogs, but also to their crosses and mongrels, since treatment of a deletion carrier with these compounds may lead to its death.

In this study, the allelic frequencies of nt230(del4) MDR1 mutation in affected breeds, their crosses, unrelated pure breeds and mongrels are reported for the state of Israel (*n* = 1416 dogs). The Israeli data were compared with reports from the US, Europe, UK, Australia and Japan.

**Results:**

The allelic frequencies of nt230(del4) MDR1 mutation in Israel for Australian, Swiss and German Shepherds (31%, 17% and 2.4%, respectively) are similar to the corresponding frequencies worldwide, much higher for Border Collies (4.8%), twice lower for Rough Collies (28%, compared to 55% or more elsewhere), and ~1% for mongrels. The frequencies for crosses of Australian Shepherd and Border Collies in Israel are 4 and 1.6 times lower, respectively, compared to the frequencies for the respective pure breeds.

**Conclusions:**

This work, that for the first time presents the frequency of nt230(del4) MDR1 mutation in Israel, along with a worldwide survey, has implications for clinicians, owners and breeders of sheepdogs and their crosses and supports the need for extra care in treatment and in future breeding. Of note, the relative proportion of affected breeds, in the overall tested dogs, might be higher than their actual proportion in Israel due to directed samples collection by veterinarians for clinical purposes, as these are mainly limited to certain affected breeds or dogs that resemble them.

**Electronic supplementary material:**

The online version of this article (10.1186/s12917-017-1251-9) contains supplementary material, which is available to authorized users.

## Background

The multi drug resistance 1 (MDR1) gene, a member of the ATP-binding cassette (ABC) transporters superfamily, is a highly conserved ATP-dependent P-glycoprotein (P-gp) membrane transporter [1]. In eukaryotes, the extrusion transporters have a fundamental function as eliminators of several toxic compounds, mainly hydrophilic and amphiphatic ones [2–4]. ABCB1 (ABC subfamily B member 1, also referred to as MDR1 and PGY1) transporters are expressed in several organs and their role in the gut epithelia and the blood-tissue barriers (blood-brain barrier, blood-testis barrier and placenta) of mammals has been studied extensively [5]. While MDR1 activity in the small intestine can limit the bioavailability of various drugs, its inactivity in the luminal membranes of the endothelial cells of the brain can lead to toxic accumulation of xenobiotics in the central nervous system (CNS) and to severe adverse effects, including death [6, 7].

Since the early 1980s, sheepdogs were reported to have sensitivity to ivermectin, a relatively new macrocyclic lactone parasiticide drug [8, 9]. Elevated concentrations of ivermectin were found in the CNS of affected dogs [10]. In 2001, ivermectin sensitivity in Rough Collies was associated with a 4-base pair (bp) deletion mutation in the fourth exon of the canine ABCB1 gene [11]. The deletion, usually annotated *ABCB1:c.227_230delATAG* or ABCB1–1∆ (MDR1) mutation (hereafter nt230(del4) MDR1 mutation), causes a frameshift that leads to a premature stop codon and a truncated transporter of 91 amino acids as opposed to 1281 amino acids, i.e. only 7.1% of the full-length mature P-gp protein [12]. Later studies showed that nt230(del4) MDR1 mutation heterozygous dogs can be regarded as having an intermediate macrocyclic lactone sensitive phenotype. This is a relevant clinical notion in cases of high-dose protocols [6, 13]. Pertinent macrocyclic lactones include ivermectin, doramectin, moxidectin and milbemycin oxime. Other drugs commonly used in the veterinary clinic, such as P-glycoprotein substrates of the non-macrocyclic lactones type, were also reported as toxic to nt230(del4) MDR1 mutation homozygote dogs (−/−). These include vincristine, digoxin, mexiletine, quinidine, fexofenadine, vinblastine, and loperamide, cyclosporine A, verapamil, paclitexal, doxorubicin, dexamethasone and others [14–19]. Moreover, treating nt230(del4) MDR1 mutation heterozygote dogs (+/−) with reduced doses of P-glycoprotein substrates such as vincristine, vinblastine and doxorubicin (but not a full dosage treatment of a non-P-glycoprotein substrate) may cause delayed drug excretion, and consequently drug toxicosis and myelosuppression [20]. Recently, a collie affected (homozygote) by the Nt230(del4) MDR1 mutation mutation experienced exaggerated CNS depression when treated with apomorphine [21], an opioid which has not been described as a P-glycoprotein substrate. Yet, other opioids, including loperamide and butorphanol, are known substrates for canine P-glycoprotein [22]. A cell line for assessing drugs as canine P-gp substrates was recently developed [23]. Noteworthy, based on its anti-parasitic and anti-inflammatory activities, ivermectin has recently received US Food and Drug Administration (FDA) and EU approval for the treatment of adult human patients for a growing number of indications [24].

The nt230(del4) MDR1 mutation has to date been detected in diverse dog breeds, including Rough Collies and other related sheepdogs, such as Australian Shepherds, miniature Australian Shepherds, Border Collies, Shetland Sheepdogs, Old English Sheepdogs, English Shepherd, German Shepherd, White Swiss Shepherd, McNab as well as Wäller and two sighthounds, Longhaired Whippet and Silken Windhound. From a genealogy perspective, all of the above breeds share a common ancestor that lived in Great Britain before the breeds were classified and registered in 1873 [15, 25]. This notion is consistent with genomic data that cluster these breeds in common clusters or sometimes even fail to differentiate between close varieties that may have undergone recent selection based on size, such as in the case of Rough Collies and Shetland Sheepdogs [26, 27].

A fast and simple detection method using allele-specific loop-mediated isothermal amplification (AS-LAMP) [28] as well as PCR-based diagnostic tests [29, 30] and the TaqMan allelic discrimination assay [15] were designed as a result of the high clinical importance of nt230(del4) MDR1 mutation detection. Efforts to map allele frequencies in affected pure breed dogs, some of their crosses, and unrelated purebred dogs have been made in different parts of the world [19, 25, 31–40]. These studies supply clinicians with regional information on the mutation distribution in their countries and highlight the need for a genetic test prior to any treatment involving common parasiticides in affected breeds and their crosses.

In Israel, spirocercosis is a widespread disease and dogs are routinely treated with macrocyclic lactones parasiticide. On average, more than 20 dogs are diagnosed annually, with the highest infection rates in the center of the country [41, 42]. The aim of this study was to determine the frequency of the mutant allele in Israel in the affected breeds, their crosses and undefined dogs, and to compare the data with recent worldwide studies. To the best of our knowledge, this is the first report on nt230(del4) MDR1 mutation frequency in Israel and it may provide a comparative perspective. It should be noted that the evaluation of nt230(del4) MDR1 mutation genetic disposition in diverse canine breeds was performed in order to inform medical and clinical professionals, rather than to illuminate the relationship of different genotypes with the clinical signs in each breed. The latter is not within the scope of this study.

## Methods

### Animals

Biological samples from 1416 dogs (blood, blood on paper and hair roots) were voluntarily and non-selectively obtained from veterinarians, breeders and owners from all regions of the country, and analyzed for the nt230(del4) MDR1 mutation as part of the diagnostic research service at our institute. A total of 481 samples from 7 purebred breeds known to bear the nt230(del4) MDR1 mutation allele (including 5 crosses of two of these breeds) and 280 crosses of these same breeds (dogs for which one parent was known to be a purebred dog, often either Australian Shepherd or Border Collie), 61 unrelated purebred dogs and 594 unclassified crosses (mongrels, dogs for which either both parents were known to be non-purebred dogs or the parentage was unclassified) were obtained. Breed status was reported by the veterinarian or owner, and was not confirmed by other inspection.

### DNA extraction

DNA from blood and blood on paper samples was extracted with the Chelex method using Bio-Rad, ‘InstaGeneTM Matrix’. Briefly, 5 μL of whole blood (or 0.25 cm^2^ paper with blood) were incubated at room temperature for 15–30 min and then centrifuged at 12,000 rpm for 3 min. The supernatant was carefully withdrawn and 200 μL of InstaGene matrix were added to the pellet and incubated at 56 °C for 15–30 min, vortexed and incubated in a 100 °C heat block for 8 min. After incubation, spin down was performed at 12,000 rpm for 3 min. The resulting supernatant contained the DNA. DNA from hair roots was extracted with the nexttec ‘Special Protocol DNA Isolation Hair by nexttec™ 1-Step’. Briefly, three to five hair roots were dissolved with Proteinase K and DTT. Samples were then incubated in a shaker (at 56 °C, 200 rpm for 2 h). Then, 100 μL of the lysates were incubated for 3 min at room temperature and later centrifuged and eluted at 700×g for 1 min. All procedures were performed according to these kits’ extraction manuals and according to the facility guidelines.

### Genotyping by Taqman assay

nt230(del4) MDR1 mutation was screened using the Fluidigm BioMark apparatus (Fluidigm Corporation US) with an Assay-specific TaqMan fluorescence probe mix. The probe sequences, which were designed following NC_006596.3, are as follows: VIC Probe Sequence was ATGACAGATAGCTTTGCAA (wt), FAM Probe Sequence AACATGACAGCTTTGCAAA (mutant); the forward and reverse primers are CCATCATCCATGGAGCTGC and CACAAATAATACTTACTTTCATTAATTATAACTGG, respectively, amplicon size 133 bp. Assay along with PCR master mix were run in duplicate by loading 5 μl into each well of the primed 96.96 Fluidigm Chip. The chip was then placed in the integrated fluidic circuit controller and loaded before analysis with the BioMark reader. The following thermal cycling protocol was used: 50°C (2 min), 70°C (30 min), 25°C (10 min), 50°C (2 min), and 95°C (4 min). This was followed by 40 cycles of 95°C (10 s) and 61°C (30 s). The initial cycle [50°C (2 min), 70°C (30 min), 25°C (10 min)]. Data were analyzed and cycle threshold (CT) values were determined using BioMark real-time PCR analysis software (Fluidigm Corp.), and automated mutation calling was carried out using an algorithm based on the change in CT (DCT) values between the wild-type and the mutant.

### Validation by PCR and Sanger sequencing

Each nt230(del4) MDR1 mutated allele was validated by PCR and Sanger sequencing. The nt230(del4) MDR1 mutation region was amplified using a touchdown reaction (4 cycles: 94°C (30 s), 58°C (30 s), 72°C (30 s); 30 cycles: 94°C (30 s), 56°C (30 s), 72°C (30 s)) with the following forward and reverse primers: TTTTTAGTTTCGCTATTCAAATTGGC and CAAACTTATTACCAATATTAACTGTAGCTC, respectively. Sequencing reactions for both DNA strands were performed with BigDye Terminator Cycle Sequencing Ready Reaction Kit (Applied Biosystems, Foster City, CA) and analyzed with an automatic sequencer (Applied Biosystems).

### Data analysis

Breeds were classified into two main groups according to nt230(del4) MDR1 mutated allele breed genealogy: Related (as detailed in the introduction) and non-related (all others). Within each group, breeds were subsequently separated into pure breeds and mix breeds. Worldwide mapping of the allelic frequency of nt230(del4) MDR1 mutation only includes breeds for which at least 50 dogs were genotyped.

## Results and discussion

### nt230(del4) MDR1 mutation allelic frequencies in Israel

Raw data is provided in Additional file 1 (for each sample ID, the breed and MDR1 genotype status are indicated). Table 1 summarizes the allelic frequencies of the canine nt230(del4) MDR1 mutation in Israel in all mentioned subgroups. The nt230(del4) MDR1 mutation frequency varied markedly by breed. Among the relatively abundant breeds (sample size greater than 10 dogs, not including 37.5% for Old English Sheepdog where the sample size was only 4 dogs), it was highest for Australian Shepherds (31.4%, where 9.0% of 145 dogs were homozygotes for the mutation), Rough Collies (27.5%, *n* = 20 and 5.0%, respectively), and Shetland Sheepdog (26.9%, *n* = 13 and 0%, respectively), followed by White Swiss Shepherd (16.7%, *n* = 12 and 8.3%, respectively), Border Collie (4.8%, *n* = 269 and 2.3%, respectively), and German Shepherd (2.4%, *n* = 21 and 0%, respectively). As expected, an intermediate frequency (10.0%) was observed in crosses of pure Australian Shepherds and pure Border Collies (*n* = 5). The respective allelic frequencies among crosses of these breeds were much lower compared to their purebred counterpart, as expected: 4 and 1.6 times lower in Australian Shepherd (7.8%, *n* = 32) and Border Collie (3.0%, *n* = 183), respectively. The nt230(del4) MDR1 mutation allele was not detected in any of the unrelated purebred dogs (*n* = 61), as expected [43]. Almost 2% (*n* = 594) of the mongrels are affected (heterozygotes for the mutation, allelic frequency of almost 0.9%), a finding that justifies a more cautious approach in the clinic in case of unspecified dogs. This value is at the lower end of the previously reported 2–7% [32, 37].

**Table 1:** Observed frequencies of nt230(del4) MDR1 mutation in related (affected known breeds, pure and crossed ones), and non-related (un-affected known breeds and mongrels) breeds in Israel

nt230(del4) MDR1 mutation allele breed genealogy	Breed			Frequency of nt230(del4) MDR1 mutation allele status [%]			
	Name	Status	Sample size	Homozygote (−/−)	Heterozygote (+/−)	Normal (+/+)	Frequency of nt230(del4) MDR1 mutation allele
Related	Australian Shepherd	Pure	145	9.0%	44.8%	46.2%	31.4%
	Australian Shepherd	Crosses	32	0.0%	15.6%	84.4%	7.8%
	Border Collie	Pure	261	2.3%	5.0%	92.7%	4.8%
	Border Collie	Crosses	183	0.0%	6.0%	94.0%	3.0%
	Border Collie X Australian Shepherd	Pure	5	0.0%	20.0%	80.0%	10.0%
	Rough Collie	Pure	20	5.0%	45.0%	50.0%	27.5%
	Rough Collie	Crosses	49	2.0%	4.1%	93.9%	4.1%
	German Shepherd	Pure	21	0.0%	4.8%	95.2%	2.4%
	German Shepherd	Crosses	11	0.0%	0.0%	90.9%	0.0%
	White Swiss Shepherd	Pure	12	8.3%	16.7%	75.0%	16.7%
	White Swiss Shepherd	Crosses	4	0.0%	0.0%	75.0%	0.0%
	Shetland Sheepdog	Pure	13	0.0%	53.8%	46.2%	26.9%
	Shetland Sheepdog	Crosses	1	0.0%	0.0%	100.0%	0.0%
	Old English Sheepdog	Pure	4	0.0%	75.0%	25.0%	37.5%
	Old English Sheepdog	Crosses	0	0%	0.0%	0.0%	0%
Non-Related	Others	Pure	61	0.0%	0.0%	100.0%	0.0%
	Others (mongrels)	Crosses	594	0.0%	1.9%	98.1%	0.9%

‘+’ stands for WT allele; ‘-’ stands for nt230(del4) MDR1 mutation allele

### A worldwide view – Comparative perspective

**Fig. 1:**
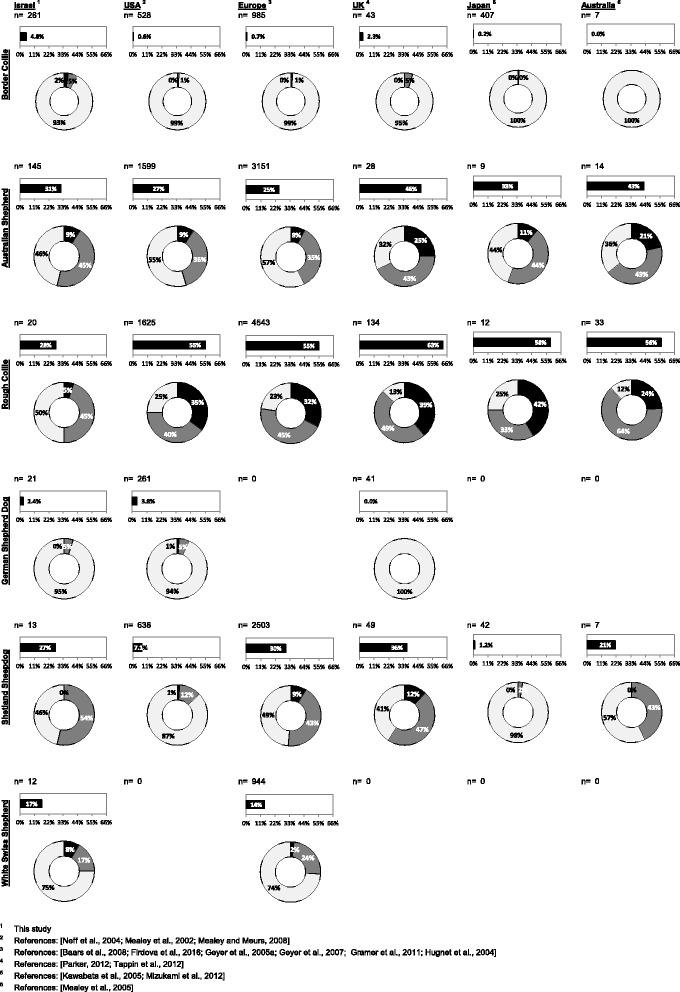
Allelic frequencies of nt230(del4) MDR1 mutation in six related breeds (from top to bottom: Border Collie, Australian Shepherd, Collie, German Shepherd Dog, Shetland Sheepdog and White Swiss Shepherd) in Israel, USA, Europe, UK, Japan and Australia (footnotes 1 to 6 respectively with the relevant literature in the bottom of the panel). Dogs number is given on top of each panel as n. Bar graph indicate allele frequency of each breed, pie graph indicate allelic frequencies of nt230(del4) MDR1 mutation as follows: pale gray for normal (+/+), gray for heterozygote (+/−) and black for homozygote (−/−)

Figure 1 presents a comparative view of the Israeli and the worldwide nt230(del4) MDR1 mutation allelic frequencies. In most cases, the frequencies in Israel seem to be similar to those in other countries, whereas in other cases the range of frequencies is wide [also see 6]. These values might also be biased by the sample size, which varied greatly in different studies, and/or by the collection method. The frequencies observed for Australian Shepherd dogs living in Israel, US, Europe and Japan are similar, ranging from 25% to 33%, while higher frequencies were observed in the UK and Australia (~45%). Allelic frequencies for Border Collie are below 1% across the world, except 2.3% in the UK, compared to a much higher frequency in Israel (4.8%). This might be an indication for over inbreeding of carrier dogs in Israel. In contradistinction, the worldwide frequencies for Rough Collies are very high (55% or higher) compared to an almost 2-fold lower rate in Israel (28%, however the sample size was small). This study provides evidence for the existence of the mutation in the popular German Shepherd, for the first time outside the US, with frequencies ranging from ~2.5% to ~4%. Similarly the nt230(del4) MDR1 mutation allele is reported in White Swiss Shepherd outside Europe, for the first time, with a similar frequency in Israel (~15%). These two latter pure breeds, the German Shepherd and the White Swiss Shepherd, arise from the same lineage, as older breed standards of the German Shepherd allowed all color varieties, including white [38]. The differences in allelic frequencies between these breeds might indicate over inbreeding in the case of the White Swiss Shepherd or for a founder effect in the breeds founding stock, back in the 1960s and 1970s [38]. The allelic frequency observed for Shetland Sheepdogs in Israel is very similar to that in Europe (27% and 30%, respectively), close to that in Australia (21%) and the UK (36%), while the frequencies in the US (7.1%) and Japan (1.2%) are much lower.

It is pertinent to state that the relative proportion of affected (predominantly pure) breeds, in the overall tested dogs in this study, might be higher than their actual proportion in Israel. The bias is related to samples collection by professional veterinarians for clinical purposes, as these are mainly limited to certain affected breeds, or dogs that resemble these breeds, from specific areas of the country where spirocercosis is more widespread [41]. Nevertheless, the allelic frequency of nt230(del4) MDR1 mutation within each affected breed is probably not significantly (if at all) affected by this bias, while it can be affected in the non-related breeds.

## Conclusions

Here, we report for the first time on the allelic frequencies of the nt230(del4) MDR1 mutation in affected breeds, as well as in their crosses and even in mongrels, in Israel. The Israeli data was compared to a worldwide literature survey which summarizes the corresponding allele status in the USA, Europe, UK, Australia and Japan. The main findings are:

- nt230(del4) MDR1 mutation allele frequencies in Israel resemble, per breed, the status in the world. Exceptions to this finding are the Israeli Border Collies and Rough Collies which have the highest (4.8%) and lowest (28%) frequency rates in the world.
- The first report on the existence of the nt230(del4) MDR1 mutated allele outside the USA and Europe on German Shepherd Dogs and White Swiss Shepherds, respectively.
- High prevalence rate of the nt230(del4) MDR1 mutated allele in mixes of prone breeds.
- The nt230(del4) MDR1 mutation frequency in dogs which were described as unrelated mongrels is 1%.

Noteworthy, due to inherent bias in the sample collection by veterinarians for clinical purposes, the relative proportion of affected breeds, in the overall tested dogs, might be higher than their actual proportion in Israel. Nevertheless, the allelic frequency of nt230(del4) MDR1 mutation within each affected breed is probably not significantly affected by this bias, while it can be affected in the non-related breeds.

The allelic frequencies of the nt230(del4) MDR1 mutation in affected breeds seems to vary slightly across the world. This notion is in agreement with the strict registry breeding procedures in breeding clubs that do not permit “new genes” to enter the breeding stock. The breeding aims at meeting the breed standard, which is a combination of phenotypic and characteristic traits with less emphasis on genetic predisposition for ailments.

This work, that for the first time presents the nt230(del4) MDR1 mutation frequency status in Israel, along with a worldwide survey, has implications for clinicians, owners and breeders of sheepdogs and their crosses and supports the need for extra care in treatment and in future breeding.

## Additional file

Additional file 1: List of all tested dogs, stratified by breed. For each sample ID, the breed and MDR1 genotyping status is indicated. (XLSX 60 kb)

## Abbreviations

*ABC*: ATP-Binding Cassette transporters superfamily;
*ABCB1*: ABC subfamily B member 1
*AS-LAMP*: Allele-Specific Loop-Mediated isothermal amplification
*CNS*: Central Nervous System
*FDA*: Food and Drug Administration
*MDR1*: Multi Drug Resistance 1
*P-gp*: P-glycoprotein
